# Advance in ERG Analysis: From Peak Time and Amplitude to Frequency, Power, and Energy

**DOI:** 10.1155/2014/246096

**Published:** 2014-07-01

**Authors:** Mathieu Gauvin, Jean-Marc Lina, Pierre Lachapelle

**Affiliations:** ^1^Department of Ophthalmology & Neurology-Neurosurgery, Montreal Children's Hospital Research Institute, McGill University, 2300 Tupper Street, Montreal, QC, Canada H3H 1P3; ^2^Département de Génie Électrique, École de Technologie Supérieure, Montréal, QC, Canada; ^3^Centre de Recherches Mathématiques, Montréal, QC, Canada

## Abstract

*Purpose*. To compare time domain (TD: peak time and amplitude) analysis of the human photopic electroretinogram (ERG) with measures obtained in the frequency domain (Fourier analysis: FA) and in the time-frequency domain (continuous (CWT) and discrete (DWT) wavelet transforms). *Methods*. Normal ERGs (*n* = 40) were analyzed using traditional peak time and amplitude measurements of the a- and b-waves in the TD and descriptors extracted from FA, CWT, and DWT. Selected descriptors were also compared in their ability to monitor the long-term consequences of disease process. *Results*. Each method extracted relevant information but had distinct limitations (i.e., temporal and frequency resolutions). The DWT offered the best compromise by allowing us to extract more relevant descriptors of the ERG signal at the cost of lesser temporal and frequency resolutions. Follow-ups of disease progression were more prolonged with the DWT (max 29 years compared to 13 with TD). *Conclusions*. Standardized time domain analysis of retinal function should be complemented with advanced DWT descriptors of the ERG. This method should allow more sensitive/specific quantifications of ERG responses, facilitate follow-up of disease progression, and identify diagnostically significant changes of ERG waveforms that are not resolved when the analysis is only limited to time domain measurements.

## 1. Introduction

The electroretinogram (ERG) identifies the electrical signal that is generated by the retina in response to a light stimulus. It is the first biopotential ever recorded from a human subject, namely, by Dewar in 1877 [1]. However, despite significant (r)evolution in the recording technologies (essentially from the string galvanometer to the digital amplifier and supporting computer software) and, consequently, the significantly enhanced quality of the ERG signal thus obtained, analysis of the ERG remains for the most part limited to amplitude and peak time measurements of its major components, namely, the a- and b-waves. This is at least what is recommended in the ERG standard of the International Society for Clinical Electrophysiology of Vision (ISCEV) [2]. The a- and b-waves of the ERG are said to reflect the activity generated by the photoreceptors and the bipolar-Müller cell complex, respectively [3–5]. These components are usually referred to as the slow waves of the ERG. Also identified in the ERG signal are the small, high-frequency, oscillations that are often seen riding on the ascending limb of the b-wave [6, 7]. These components, referred to as oscillatory potentials (OPs), are most probably generated by the retinal cells of the inner retina (i.e., bipolar, amacrine, or horizontal cells) although their exact origin remains debated [8, 9]. The OPs appear to be major contributors to the shaping of the ERG waveform [10] and there is an abundant literature attesting to the clinical value of including the OPs when analyzing pathological ERGs [6, 11, 12]. Unfortunately, in order to optimize the visualization of the OPs one must modify the recording bandwidth of the ERG from a broadband (e.g., 1–1000 Hz) to a narrower band (e.g., 100–1000 Hz) that removes the low-frequency components of the ERG (i.e., a- and b-waves) and consequently selectively enhances the high-frequency components (i.e., OPs) [2]. However, when doing so one must always keep in mind the possibility of introducing artifactual components (such as ringing artifacts and phase lags) to the ERG thus obtained.

It is clear from the above that the ERG waveform results from the amalgamation of several frequency components. Is it possible to monitor the frequency composition of the ERG signal without altering the signal as it is done with the bandwidth restriction approach? Would the use of such an approach significantly improve analysis of the ERG beyond what is accomplished when using time and amplitude measures of the ERG only? Although advanced analytical approaches are now frequently used when studying biopotentials, such as the electroencephalogram [13], the electrocardiogram [14], and the electromyogram [15], to date they have only been sporadically applied to the ERG [8, 16, 17]. The purpose of this study was therefore to compare peak time and amplitude measurements of human photopic ERGs with measures obtained in the frequency domain using the Fourier analysis as well as in the time-frequency domain using the continuous (CWT) and discrete (DWT) wavelet transforms. For the sake of brevity, our study was limited to the photopic ERG only.

## 2. Materials and Methods

Normal photopic ERGs were obtained from 40 healthy subjects (26 females and 14 males, average age 29.9 ± 8.4 years) using a protocol that was approved by the Institutional Review Board of the Montreal Children's Hospital and in accordance with the Declaration of Helsinki.

According to a previously published method of ours, the ERGs were recorded with both eyes dilated (tropicamide 1%) using an active electrode (DTL fiber electrode) placed in the inferior conjunctival bag, with reference and ground electrodes pasted at the external canthi and forehead, respectively [18–21]. The ERGs were evoked to flashes of white light (flash duration: 20 *μ*s; interstimulus interval: 1.5 s; and average of at least 10 flashes per recording) of 0.64 log cd*·*s*·*m^−2^ in intensity that were delivered against a rod desensitizing background light of 30 cd*·*m^−2^ (measured using a research radiometer IL1700; International Light, Newburyport, MA, USA). ERG waves from both eyes were averaged to yield a single waveform of 150 ms in length (sampling rate: 3413.33 Hz) that included a prestimulus baseline of 20 ms.

### 2.1. ERG Analysis

The amplitude of the a-wave was measured from the prestimulus baseline to the most negative trough of the ERG, while the amplitude of the b-wave was measured from the trough of the a-wave to the most positive peak of the ERG that followed the a-wave [2]. Peak times were measured from flash onset to the peak of the a- and b-waves [2]. Given that these measures of the ERG are taken in the time domain, they will be referred to as time domain (TD) measurements.

Frequency domain analysis (or Fourier analysis (FA)) of the ERG was carried out using the fast Fourier transform (FFT) algorithm implemented in MATLAB R2013b (Mathworks, Natick, MA, USA) as follows:

(1) $$\begin{array}{c} X\left(k\right)=\overset{N-1}{\underset{t=0}{\sum }}x\left(t\right)e^{-i(2\pi /N)tk},\;k=0,1,\ldots ,N-1, \end{array}$$

where *X*(*k*) represents the FFT coefficients, *x*(*t*) denotes the raw ERG time-series, and *N* denotes the number of data points in *x*(*t*). Each FFT coefficient weighs the energetic contribution of a single frequency component to the signal so that a frequency spectrum can be illustrated by tracing *X*(*k*). Considering the size (512 data points) and sampling frequency (3413.33 Hz) of our ERG waveforms, we were able to compute the FFT coefficients for frequencies ranging between 0 and 1706.66 Hz in increments of 6.66 Hz (i.e., frequency resolution). However, given the limitation imposed by our recording bandwidth (1–1000 Hz) we limited our analysis to frequencies ranging between 0 and 300 Hz to safely avoid artifactual contamination (such as that predicted by the Nyquist-Shannon sampling theorem [22]).

In order to localize the energy content of the ERG in both time and frequency we computed, using MATLAB, the continuous wavelet transform (CWT) of selected ERGs as follows:

(2) $$\begin{array}{c} \text{CWT}\left(a,b\right)=\frac{1}{\sqrt{a}}\overset{+\infty }{\underset{-\infty }{\int }}x\left(t\right)\psi^{*}\left(\frac{t-b}{a}\right)dt, \end{array}$$

where CWT(*a*, *b*) represents the wavelet coefficients localized at scales *a* (frequency) and moments *b* (time), *x*(*t*) denotes the unprocessed ERG time-series, and ψ* denotes the complex conjugate of the Morse wavelet [23], which was chosen for its good frequency resolution [24]. To illustrate the time-frequency scalogram of the CWT, we took the absolute value of CWT(*a*, *b*) and normalized it to its maximal value so that the time-frequency scalograms of the ERG are shown as colored two-dimensional plots of CWT(*a*, *b*) in which minimum energy values are displayed in blue and maximal values in red.

Use of the CWT approach allowed us to analyse the ERG, continuously, at every possible scale *a* and translation *b*. This approach, however, requires extensive computation time and also yields a lot of redundant information (i.e., since each coefficient has similar neighboring values) that will remain unused in the set of coefficients CWT(*a*, *b*) [25]. Interestingly, if the scale and translation parameters of the wavelet are taken at discrete values, we then obtain a discrete wavelet transform (DWT), where the scales *a* and translations *b* are based on powers of two (i.e., *a*_*j*_ = 2^*j*^, *b*_*j*,*k*_ = *k*2^*j*^) so that (2) can be discretized as follows:

(3) $$\begin{array}{c} \text{DWT}\left(j,k\right)=\overset{+\infty }{\underset{-\infty }{\int }}x\left(t\right)2^{-j/2}\psi \left(2^{-j}t-k\right)dt, \end{array}$$

where DWT(*j*, *k*) represents the wavelet coefficients localized at discrete scales *j* (frequency) and discrete moments *k* (time), *x*(*t*) designates the raw ERG time-series, and *ψ* designates the Haar wavelet [25]. DWT(*j*, *k*) was computed using the fast wavelet transform algorithm of Mallat [25, 26] implemented in MATLAB. We chose the Haar wavelet, for its simplicity (i.e., simplest wavelet available) and its orthonormal basis, which allows the wavelet coefficients DWT(*j*, *k*) to be reconstructed accurately and efficiently without any loss of information (using the inverse DWT) even if all the redundant information contained in the CWT is discarded [25, 27]. Similar to the CWT scalograms, the most prominent energy component of the DWT scalogram will appear as a deep dark red (high energy) rectangle in the region of the DWT scalogram where it is located (i.e., located in time and frequency), and, conversely, the absence of any component at given locations will appear as deep dark blue (no energy) rectangles.

### 2.2. Statistical Analyses

Mean value, standard deviation (SD), and coefficient of variation (CV) were computed for all ERG parameters that were identified using the different analytical approach. *Z*-scores were used to evaluate the significance of selected descriptor changes. All tests were set to a level of significance of 5%.

## 3. Results

### 3.1. Time and Amplitude Measurements in the Time Domain

**Figure 1:**
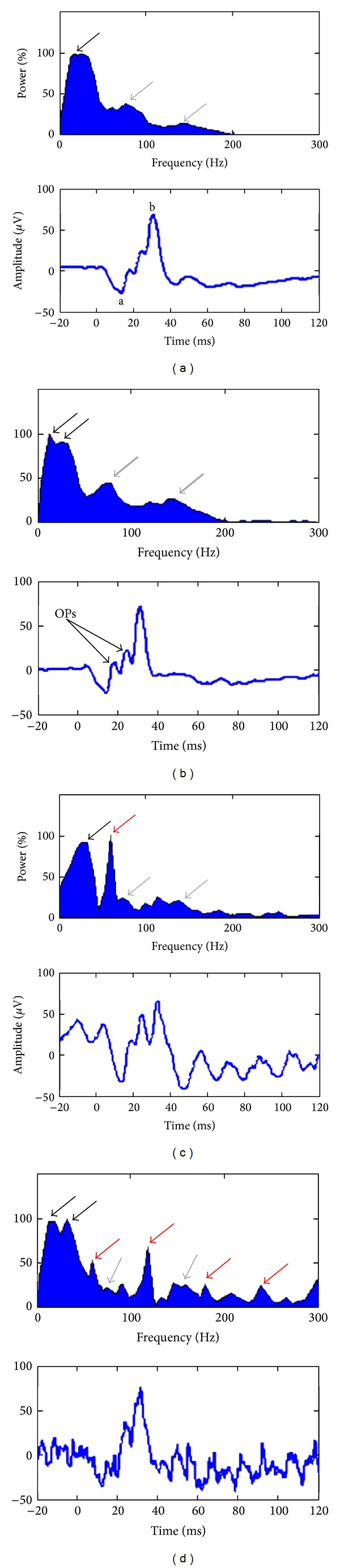
Fourier analysis (FA) of 4 normal ERGs. The frequency spectrums are shown as normalized power spectrum, in percentage, where the spectrums are normalized to their maximal value. The associated ERGs are shown at the bottom of each spectrum. The a-wave, b-wave, and OPs are indicated as “a,” “b,” and “OPs,” respectively. (a) FA of a composite ERG, averaged from 40 subjects, showing the 3 typical frequency components that contribute to the ERG (~30 Hz: a- and b-waves contribution, black arrow; ~75 Hz and ~150 Hz: oscillatory potentials (OPs) contribution, gray arrows). (b) FA of an ERG showing enhanced OPs (increased ~75 Hz and ~150 Hz component contribution; thick gray arrows). (c) FA of a typical contaminated ERG showing the 3 standard ERG components (see arrows, same color-coding as previous panels) and a sharp, noise-related, maximal component at 60 Hz (60-cycle line interference contribution, red arrow). This sharp noise component seems to disturb the identification of the OPs component located at ~75 Hz. (d) FA of another contaminated ERG showing the 3 characteristic frequency components (see arrows, same color-coding as previous panels) of the ERG and 4 interference-related components at 60, 120, 180, and 240 Hz, respectively (60-cycle harmonics contribution, red arrows). These noise components seem to complicate the identification of the two typical OPs components located at ~75 Hz and ~150 Hz.

As reported in Table 1, time domain analysis allowed the identification of two major ERG components, one peaking at 13.53 ± 1.55 ms (mean ± SD obtained in our 40 subjects) with an amplitude of 32.21 ± 5.11 *μ*V (identified as the a-wave at Figure 1(a)) and another one which peaks at 30.98 ± 1.33 ms with an amplitude of 104.81 ± 18.66 *μ*V (identified as the b-wave at Figure 1(a)).

### 3.2. Fourier Analyses (FA)

The frequency components contributing to the genesis of the ERG can be identified using the FA, such as that obtained with the FFT. This is best exemplified in Figure 1 and Table 1, where three major frequency components are identified in the normal ERGs. As shown with the black arrows, the low-frequency components of the ERG (presumably a- and b-waves) usually formed a smooth peak of large magnitude, culminating at 28.8 ± 5.7 Hz (i.e., mean ± SD obtained from our 40 subjects) on the frequency spectrum. However, in some instances, two distinct peaks could be identified (see double black arrows in panels (a) and (d)). In the later cases, only the peak of highest magnitude was considered for further analysis. In contrast, the higher frequency components of the ERG (probably the OPs) usually formed two distinct peaks (see grey arrows) of low magnitude located at 75 ± 7.7 Hz and 146 ± 13.3 Hz, respectively. However, given that FA only looks at the frequency content of the ERG without taking into consideration if those frequencies are time-locked or not to the stimulus, its use can lead to erroneous interpretations of the ERG. This is best illustrated with the ERGs shown in Figures 1(c) and 1(d) where the noise contaminants (such as 60 Hz in Figure 1(c)) appear to contribute more to the making of the ERG than the retinal evoked components themselves. These limitations of FA can be overcome by adding a temporal resolution to the frequency domain.

### 3.3. Continuous Wavelet Transform (CWT) Analyses

**Figure 2:**
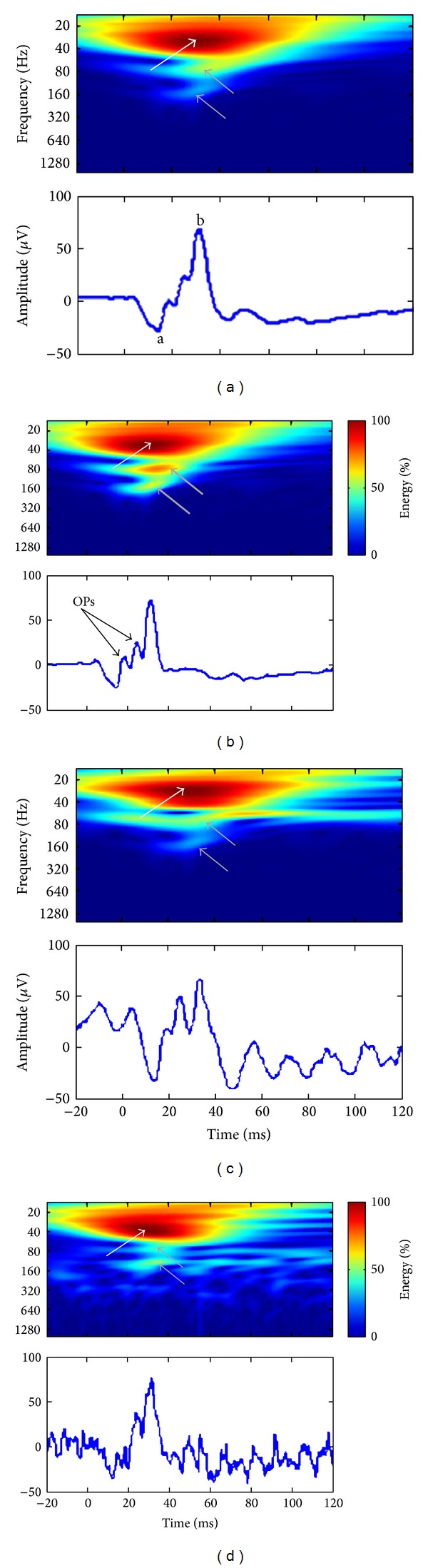
Continuous wavelet transform (CWT) analysis of the ERGs that were shown at Figure 1. All scalograms were normalized to their maximal energy values, and the color-coding (see colorbar) indicates low (blue), moderate (green), and high (red) energy values. The associated ERGs are shown at the bottom of each scalogram. The a-wave, b-wave, and oscillatory potentials are indicated as “a,” “b,” and “OPs,” respectively. (a) CWT of the composite ERG showing the energy (as per colorbar) and the temporal location of the 3 typical frequency components that were previously identified in the Fourier domain (~30 Hz: a- and b-waves contribution, white arrow; ~75 Hz and ~150 Hz: oscillatory potentials (OPs) contribution, gray arrows). (b) CWT of the ERG that had enhanced OPs [increased ~75 Hz and ~150 Hz energy; thick gray arrows]. (c) CWT of the contaminated ERG showing the 3 standard components (see arrows, same color-coding as previous panels) and a strip of moderate energy localized at 60 Hz for the whole duration of the signal (60-cycle interference contribution). (d) CWT of the second contaminated ERG also evidencing the 3 characteristic frequency components of the ERG and several transient, interference-related, patches of energy (60-cycle harmonics contribution).

As shown in Figure 2, use of the CWT approach allowed us to more precisely localize (in both time and frequency, as reported in Table 1) the abovementioned frequency components of the ERG signal and even in the presence of significant noise contaminants (as seen in panels (c) and (d)). In each scalogram, the a- and b-waves formed a cluster of hot (dark red) coefficients (see white arrows) centered at 29.7 ± 5.7 Hz (i.e., mean ± SD obtained in our 40 subjects) and peaking at 31.0 ± 0.9 ms (i.e., time-locked to the peak time of the ERG b-wave). Similarly, the OPs formed two distinct clusters (see grey arrows) centered at 73.8 ± 7.7 Hz and 150.3 ± 11.6 Hz and peaking at 32 ± 2.2 ms and 29.8 ± 1.9 ms, respectively. As shown in the scalogram of panels C and D, the high- (i.e., OPs) and low-frequency (i.e., corresponding to the a- and b-waves) components continued to remain the major light-evoked (i.e., time-locked to the stimulus) components of the ERG response in spite of significant noise contamination. The latter contrasts with results obtained using the FA where one cannot dissociate evoked from nonevoked frequency components (compare results shown in Figures 2(c) and 2(d) with corresponding FA results shown in Figures 1(c) and 1(d)).

In each scalogram of Figure 2, the value of the coefficients that identified the hot clusters pertaining to the a- and b-waves or OPs was centered on a section of the clusters where all coefficients had the same value (i.e., equipotential regions). This attribute of the CWT will generate redundant information (i.e., similar or equal coefficient values) that will complicate the accurate identification of the time-frequency coordinates (i.e., ill-posed coordinates) of the ERG components, by introducing an uncertainty factor. For example, given that the red clusters of Figure 2 extend over a large area of the scalograms, this prevents an accurate quantification of the a-wave energy, which is most probably hidden by the higher energy b-wave. These limitations can be overcome if we impose a discretization over the possible frequencies and times at which the information is computed.

### 3.4. Discrete Wavelet Transform (DWT) Analysis

**Figure 3:**
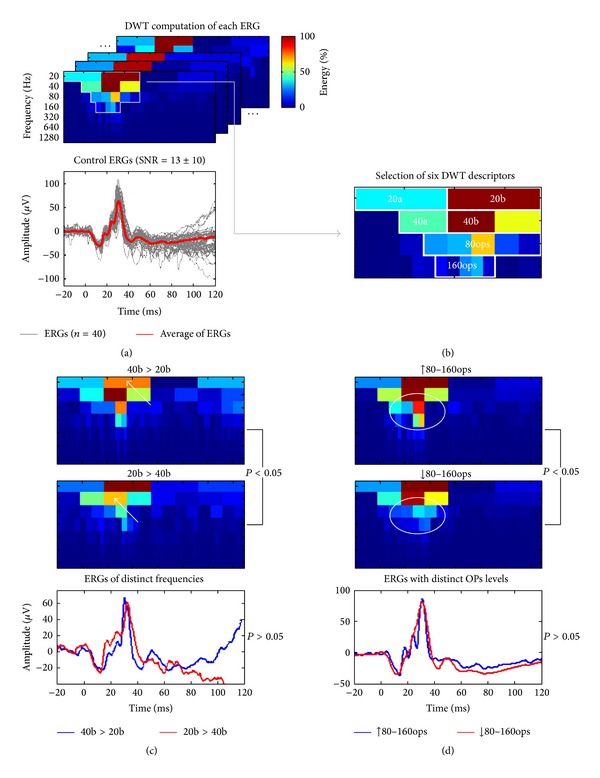
Analysis method and classification improvement of normal ERGs using the discrete wavelet transform (DWT). (a) We computed the DWT of 40 normal ERGs with various signal-to-noise ratios (SNR = 13 ± 10, gray traces; averaged response: red trace) and extracted, in each scalogram, descriptors (see panel (b)) localized inside the region of maximal energy (delimited by white borders). (b) Magnification of the averaged ERG (red trace at panel (a)) scalogram that shows where we identified six novel DWT descriptors of the ERGs (20a and 40a: a-wave energy; 20b and 40b: b-wave energy; and 80ops and 160ops: oscillatory potentials (OPs) energy). ((c) and (d)) The DWT descriptors were used to segregate ERGs of distinct morphologies, that had similar (*P* > 0.05) a- and b-wave amplitudes and peak times but significantly (*P* < 0.05) smaller 20b or 40b descriptors (panel (c), white arrows) or significantly larger or smaller 80ops and 160ops descriptors in the DWT scalograms (panel (d), white circles).

As illustrated in Figure 3, the DWT scalograms decomposed the signals into seven contiguous frequency bands (20, 40, 80, 160, 320, 640, and 1280 Hz), each including a range of frequencies (reported in Table 1) around their respective central frequency (CF). For example, the 20 Hz band quantified the energy oscillating between 13.33 and 26.66 Hz (i.e., 20 ± 20/3, that is, CF ± CF/3). Use of this expansion simplifies the choice of relevant coefficients (i.e., seen as rectangles of different sizes in the scalograms of Figure 3) that may be used as energy descriptors of the ERG.

#### 3.4.1. Identification of DWT Descriptors

As seen with the DWT scalograms of Figure 3, the major frequency components are confined to the time-frequency region that is surrounded by white borders and where six major components can be identified (i.e., rectangles of various colors shown in panel (a) and magnified in panel (b)) and thus quantified (as reported in Table 1). The DWT also removed the redundancy so that the b-wave energy, quantified with the 20b and 40b descriptors (panel (b)), is now represented by two rectangles (i.e., identified as 20b and 40b) confined to an area of the scalogram that is limited to the vicinity of the b-wave peak rather than spread across most of the CWT scalogram. This allowed the quantification of two descriptors, time-locked to the a-wave, which we identified as 20a and 40a. The 20a and 40a were of lower energy compared to the 20b or 40b (see Table 1), indicating that, as expected, the b-wave energy is greater than that of the a-wave. Finally, the high-frequency components, indicated as 80ops and 160ops, were also easily identified (i.e., maximal value in the 80 and 160 Hz bands, resp.).

#### 3.4.2. Improvement of ERG Segregation Using the DWT

As indicated above, the a- and b-wave components were seen on both the 20 Hz (20a and 20b descriptors) and 40 Hz bands (40a and 40b descriptors). These descriptors can be used to segregate ERGs of different morphologies. This is better illustrated in Figure 3(c), where two ERGs of distinct morphologies were similar (*P* > 0.05) in terms of a- and b-wave peak time and amplitude but were significantly different (*P* < 0.05) on the basis of their DWT b-wave descriptors (20b and 40b). In one example, the ascending limb of the b-wave has a sharp morphology (blue tracing) and showed a lower 20b descriptor (*P* < 0.05), compared to the broader ascending limb of the b-wave (compare first half of ascension of tracing in red) which disclosed an attenuated 40b parameter (*P* < 0.05). Similarly, we were also able to segregate ERGs that differed in OPs prominence (such as what is shown in Figure 3(d)). Although these ERGs were indistinguishable (*P* > 0.05) on the basis of peak time and amplitude measurements of the a- and b-waves, they were significantly different (*P* < 0.05) on the basis of their 80ops and 160ops energy content which were higher (blue tracing) or lower (red tracing) compared to average.

### 3.5. Applications of Refined Analytical Approach to Clinical ERGs

**Figure 4:**
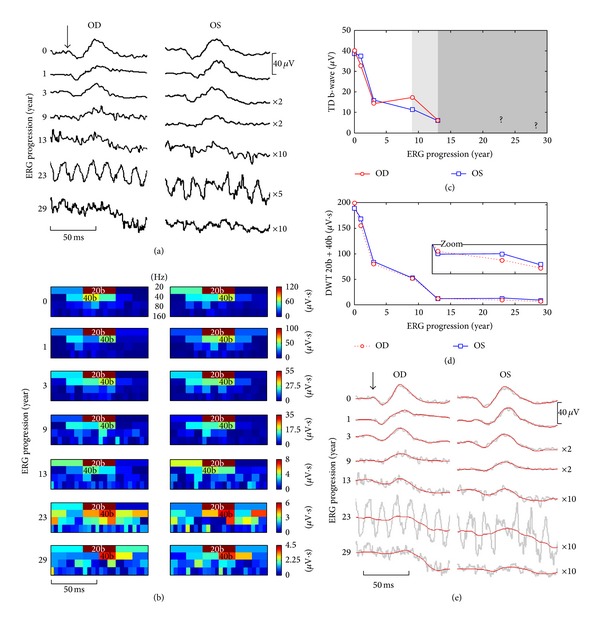
(a) ERG traces (averaged from up to 100 responses) obtained at seven time points in the right (OD) eye and left (OS) eye of a male patient affected with retinitis pigmentosa (both eyes presented with nonrecordable scotopic ERGs, constricted visual fields, pigmentary deposits, and decreased visual acuity) in a time span of 3 decades. The horizontal (time) and vertical (voltage) scale bars apply to both eyes and some traces have been magnified (×2, ×5, or ×10 times) for visualization purposes. The flash onset is indicated by the black vertical arrow. ERG progression is shown in years since the first visit on the left-hand side. (b) Scalograms computed for each pathological ERG waveform (presented in the same order than in panel (a)) in which we quantified the 20b and 40b descriptors. Note that, in some scalograms, the position of the 40b descriptors was delayed (i.e., delayed latency of the b-wave) compared to normals (see Figure 3) and the 40b descriptors were always more severely attenuated than the 20b. (c) Progression of the TD b-wave amplitudes from both eyes. Because of the noise contaminants the 4th and 5th ERG were imprecisely measured (indicated by the lighter gray background on the graph), while the last two ERGs were nonmeasurable, thus preventing the quantification of disease progression from that point (indicated by the darker gray background). (d) Using the DWT descriptors of the b-wave (20b + 40b) allowed us to monitor the disease progression more precisely and for the whole time span (additional 16 years of monitoring: see zoomed box). (e) Using the inverse DWT, we reconstructed the low-frequency bands (i.e., 20 and 40 Hz bands), obtaining the biological denoised responses which are shown, in red, on top of the unprocessed gray tracings.

**Figure 5:**
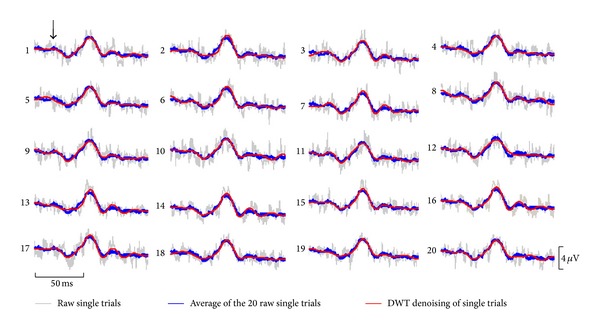
20 consecutive single-flash ERG responses (gray traces) obtained from a patient affected with retinitis pigmentosa. The average of these 20 raw ERG responses canceled the uncorrelated noise to yield the blue tracing (overlaid on top of each single-flash response). DWT denoising of the individual noisy single-flash responses reveals denoised biological responses, which are shown as red traces. All red traces are nearly identical (shape and amplitude) to the trace obtained from the average (i.e., blue traces) of the 20 noisy responses, thus validating this denoising approach. The horizontal (time) and vertical (voltage) scale bars apply to each trace. The flash onset is indicated by the black vertical arrow.

In Figure 4(a) the ERG waveforms obtained from a patient diagnosed with retinitis pigmentosa (RP) that was followed up for more than 30 years are illustrated. As shown, the low amplitude (and low signal-to-noise ratio or low SNR) of these pathological ERGs, especially those obtained later in the disease process (tracings 23 and 29), seriously compromises an accurate measurement of these waveforms. This is best exemplified in Figure 4(c), where an accurate measurement of the b-wave amplitude could only be achieved for ERGs obtained within the first 13 years, due to the highly contaminated ERGs recorded subsequently. However, use of the DWT still permitted the extraction of b-wave descriptors (i.e., 20b and 40b) as shown in the scalograms of Figure 4(b) and therefore allowed us to monitor progression of the disease process for an additional period of 16 years, as shown in Figure 4(d). Furthermore, as revealed in Figure 4(d), both eyes followed the same degeneration pattern, which appeared to follow an exponential decay function that correlated well with that which characterized (using b-wave amplitude measures) the first 13 years. Interestingly, the use of the inverse DWT of the low-frequency bands (i.e., 20 and 40 Hz bands) allowed us to reconstruct noise-free ERGs (Figure 4(e)), that were nearly identical in both eyes, as it was also the case for the ERGs (measurable, high SNR) recorded in earlier exams (tracings 0, 1, 3, and 9 of Figure 4(a)). The validation of this denoising approach (i.e., inverse DWT) is further demonstrated in Figure 5, where we reconstructed the 20 consecutive single-flash recordings obtained from a RP patient that were used to generate the average waveform. As shown, the 20 denoised single-flash waveforms (red tracings) are nearly identical (mean (±SD) Pearson coefficients = 0.92 ± 0.02) to the averaged response (i.e., blue tracing obtained by averaging the 20 consecutive noisy responses (gray traces)). In contrast, the mean Pearson coefficients obtained between the single sweeps and the averaged response were of 0.52 ± 0.03.

## 4. Discussion

To date, analysis of the ERG relies mostly on time domain (TD) measurements (peak time and amplitude) of its two major components, namely, the a- and b-waves. However, as shown with the examples illustrated in Figures 4 and 5 and as previously suggested [28–31], TD measurements are subjected to noise contamination. These contaminants can arise from numerous factors such as the subject (e.g., eye blinks, head/eye movements, etc.), external sources (e.g., mechanical vibrations, electromagnetic coupling with the 50/60 Hz power lines, computer monitors, electrical lighting, etc.), and, if the data is digitized, the digitization process itself (e.g., digitization artifacts, aliasing, etc.). Therefore, limiting the ERG analysis to TD measures only could jeopardize the detection of subtle functional changes.

### 4.1. From Fourier to Wavelets

FA methods, such as the one presented in Figure 1 (i.e., FFT), performed well in identifying the three major frequency components of the normal photopic ERG response. However, when a noise contaminant was distributed over the entire ERG response, the resulting frequency-power distribution was misleading (as shown in Figures 1(c) and 1(d)). This is due to the fact that the FA assumes that all the frequency components that compose a signal are periodic and, consequently, ignores the possibility that some frequencies could be found at precise poststimulus time locations only. This explains why the amplitude of a component can be over- or underestimated when relying solely on FA analysis. In other words, while Fourier analyses are well-suited to identify the frequency components that compose the ERG signal, they are of no use to determine their respective magnitude and temporal location within the signal. Such information is of crucial importance if one wishes to define the signal with all its subtleties. This can be accomplished with a time-frequency domain analysis of the ERG.

Use of the CWT (Figure 2) approach allowed us to clearly identify the temporal as well as the frequency coordinates of the three major frequency components of the ERG, thus remedying the FA limitations alluded to above. We have shown that, with the CWT scalograms, each of these components was time-locked to the largest wave of the ERG (i.e., the b-wave). Furthermore, compared to corresponding FA estimates, their respective weights (i.e., relative energy levels) were also more accurately determined, even in noisy ERG recordings.

### 4.2. The DWT: An Optimal Compromise

Interestingly, it seems, from FA and CWT analyses, that the components of the ERG cannot be associated with single frequency values but rather to a range of values. For example, in the FA power spectrums of Figure 1, each component had a broad Gaussian-like distribution (i.e., suggestive of a band of frequencies) rather than a sharp peak (i.e., suggestive of a single frequency) as it would be the case for a pure sinusoid. Similar broad distributions of individual frequency components were also observed in the CWT of the same ERG signals (see Figure 2), the major difference being that the magnitude of these frequency components can now be time-correlated to specific events of the ERG signal. Consequently, since these components contain a band of frequencies rather than a single frequency, analysis of the ERG at different frequency bands using the DWT scalogram (rather than at each possible frequency with the CWT) offers a simplified scheme (exempt of redundancy) to identify relevant ERG descriptors (see Figures 3(a) and 3(b)).

In the DWT scalograms the a- and b-waves were characterized by distinct components located in the 20 Hz (20a and 20b descriptors) and 40 Hz (40a and 40b descriptors) bands. It was difficult to accurately determine these distinct frequency components using the CWT, although in the FA some ERGs did show both the 20 and 40 Hz components (i.e., identified as double-peaks in Figures 1(b) and 1(d)). Furthermore, quantifying the ERG waveforms using the DWT augmented the specificity (i.e., ability to discriminate between distinct ERG morphologies, as shown in Figures 3(c) and 3(d)) and sensitivity (i.e., reduced the variability, as shown by the CV reported in Table 1) of the measures obtained.

Finally, at the end-stage of severe degenerative retinopathies (such as RP), nearly extinguished ERGs (e.g., low-SNR, such as the one shown in Figures 4(a) and 5) are often the last measurable signs of functional vision [32]. As shown in Figure 4, extracting relevant ERG descriptors from these residual ERGs using a TD approach (e.g., peak time and amplitude measures) becomes nearly impossible as the SNR decreases. Use of the DWT permitted the quantification of such responses, thus extending the length of the follow-up period of disease progression by an additional 16 years. This allowed us to demonstrate the exponential decay known to characterize the long-term course of the cone ERG amplitude in patients affected with RP [33]. Furthermore, use of the inverse DWT of selected frequency bands allowed us to reconstruct noise-free ERG waveform thus confirming the presence of a residual biological response in signals that were reported as nonmeasurable using the TD approach. The validation of this DWT-denoising approach was demonstrated in Figure 5, where each of the 20 denoised ERGs was highly similar to the averaged response (obtained by averaging the 20 consecutive responses).

### 4.3. Limitations of the Study

In this study we limited the TD approach to its most widespread descriptors (i.e., amplitude and peak time of the a- and b-waves), but other unusual descriptors (e.g., area-under-the-curve of the a- or b-wave, time to reach a certain percentage of the a- or b-wave amplitudes, steepness of the rising or descending flank of the b-wave, filtered OPs measurements, etc. [6, 31, 34]) could also be of use to identify subtle morphological changes, albeit similarly sensitive to noise contaminant errors.

## 5. Conclusions

In this paper, we have presented a brief overview of the different analytical approaches that can be used to quantify the ERG waveform. As long as the response remains measurable, the traditional measurements of the a- and b-waves can be used to monitor the peak time and the amplitude of the ERG signal. However, these measurements only look at the ERG signal as a whole, instead of looking at the different frequency components (possibly of distinct cellular origin) separately. The discrete wavelet transform offers the possibility to extract more components of the ERG signal, even in very poor SNR responses. Standardized time domain analysis of retinal function should thus be complemented with advanced DWT descriptors of the ERG. The latter should allow more sensitive/specific quantifications of ERG responses, facilitate follow-up of disease progression, and identify diagnostically significant changes of ERG waveforms that are not resolved when the analysis is only limited to time domain measurements, thus bringing the analysis and interpretation of the ERG signal in the 21st century, as it is already the case with other biopotentials such as the electroencephalogram and electrocardiogram.

## Figures and Tables

**Table 1:** Normative data (mean ± standard deviation (SD) and coefficient of variation (CV, in bold)) obtained for each parameter (time, frequency, amplitude, power, and energy) assessed using the different analytical approaches compared in this study (time domain, frequency domain, and continuous and discrete time-frequency domain). The time domain allows timing and amplitude quantification of two major components (i.e., the a- and b-waves). The frequency domain identifies the frequency and power of three major components (probably associated with the a- and b-waves and OPs). The continuous time-frequency domain allows timing, frequency, and energy measurements of three main components (probably associated with the a- and b-waves and OPs). Finally, with the discrete time-frequency domain, the components are identified in predetermined temporal windows (i.e., intervals) and frequency bands (i.e., instead of precise timing and frequency) and allow more components to be identified and the a- and b-wave can be quantified independently (i.e., in contrast to the frequency domain or continuous time-frequency domain in which the a- and b-waves formed a single low-frequency component).

Time domain (peak time and amplitude)				
	Time (ms)	Frequency (Hz)	Amplitude (*μ*V)	Origin
Main component 1	13.53 ± 1.55 **(11)**	n/a	32.21 ± 5.11 **(16)**	a-wave
Main component 2	30.98 ± 1.33 **(4)**	n/a	104.81 ± 18.66 **(18)**	b-wave

Frequency domain (Fourier analysis)				
	Time (ms)	Frequency (Hz)	Power (*μ*W/Hz)	Probable origin

Main component 1	n/a	28.81 ± 7.17 **(29)**	10.46 ± 2.15 **(21)**	a- and b-waves
Main component 2	n/a	75.38 ± 7.69 **(10)**	3.43 ± 0.89 **(26)**	OPs
Main component 3	n/a	146.00 ± 13.31 **(9)**	1.88 ± 0.43 **(23)**	OPs

Time-frequency domain (continuous wavelet transform)				
	Time (ms)	Frequency (Hz)	Energy (*μ*V*·*s)	Probable origin

Main component 1	31.04 ± 0.89 **(3)**	29.70 ± 5.71 **(19)**	202.8 ± 21.08 **(10)**	a- and b-waves
Main component 2	32.04 ± 2.22 **(7)**	73.81 ± 6.28 **(9)**	111.57 ± 23.17 **(21)**	OPs
Main component 3	29.81 ± 1.96 **(7)**	150.28 ± 11.57 **(8)**	69.29 ± 16.79 **(24)**	OPs

Time-frequency domain (discrete wavelet transform)				
	Time interval (ms)	Frequency band (Hz)	Energy (*μ*V*·*s)	Probable origin

Main component 1	−20 to 17.5	20 ± 6.6	78.08 ± 10.66 **(14)**	a-wave
Main component 2	17.5 to 55	20 ± 6.6	214.46 ± 19.02 **(9)**	b-wave
Main component 3	0 to 17.5	40 ± 13.3	100.79 ± 9.39 **(9)**	a-wave
Main component 4	17.5 to 55	40 ± 13.3	228.41 ± 28.05 **(12)**	b-wave
Main component 5	8.125 to 55	80 ± 26.6	145.83 ± 21.75 **(15)**	OPs
Main component 6	17.5 to 40.35	160 ± 53.3	80.81 ± 12.48 **(15)**	OPs
